# Synthesis of nanoporous carbonaceous materials at lower temperatures

**DOI:** 10.3389/fchem.2023.1277826

**Published:** 2023-10-13

**Authors:** Liping Zhang, Alexandre A. S. Gonçalves, Mietek Jaroniec

**Affiliations:** ^1^ Faculty of Materials Science, Shenzhen MSU-BIT University, Shenzhen, China; ^2^ Catalytic and Process Ceramics, Saint-Gobain NorPro, Stow, OH, United States; ^3^ Department of Chemistry and Biochemistry, Kent State University, Kent, OH, United States

**Keywords:** nanoporous materials, carbonaceous materials, hydrothermal carbonization, hard templating method, mechanochemical synthesis

## Abstract

Nanoporous carbonaceous materials are ideal ingredients in various industrial products due to their large specific surface area. They are typically prepared by post-synthesis activation and templating methods. Both methods require the input of large amounts of energy to sustain thermal treatment at high temperatures (typically >600°C), which is clearly in violation of the green-chemistry principles. To avoid this issue, other strategies have been developed for the synthesis of carbonaceous materials at lower temperatures (<600°C). This mini review is focused on three strategies suitable for processing carbons at lower temperatures, namely, hydrothermal carbonization, *in situ* hard templating method, and mechanically induced self-sustaining reaction. Typical procedures of these strategies are demonstrated by using recently reported examples. At the end, some problems associated with the strategies and potential solutions are discussed.

## 1 Introduction

Nanoporous carbonaceous materials (NCMs), including pure carbon, carbon materials doped with heteroatoms, or modified with functional groups, have an enormous record of important applications in our daily life and in various industries (Lee et al., 2006; Liu et al., 2015; Wang et al., 2020). Mainly because of their large specific surface area (SSA), nanoporous carbonaceous materials can be used as adsorbents in water and air purifiers, catalysts in energy-storage devices including electrochemical capacitors, batteries, solar cells, etc., and supports for emerging single-atom catalysts.

Industrial synthesis of NCMs on a large scale involves activation of bulk carbon-rich materials such as biomass and coal in the presence of corrosive substances (e.g., acids, metal hydroxides, carbonates, sulfates, etc.) and at elevated temperatures (>600°C) (Lee et al., 2006; Zhang and Jaroniec, 2020). During processing of these carbons their surface modification with functional groups is typically not considered. Bottom-up synthesis using templates allows for easy control of the composition of carbonaceous products, but it also requires calcination at high temperatures, mostly ranging from 600°C to 1,200°C (Lee et al., 2006; Libbrecht et al., 2017; Xie et al., 2020; Zhang and Jaroniec, 2020; Diez et al., 2021). Thermal treatment in both methods is accompanied by a few notable disadvantages, including 1) consumption of a large amount of heat, 2) generation of pollutants and greenhouse gases, and 3) damage of functional groups introduced in and/or on carbonaceous products as well as deterioration of porous structures. Hence, it is necessary to explore strategies for producing nanoporous carbonaceous materials at relatively low temperatures (<600°C) or even at room temperature.

Many redox reactions that create carbon as a product can be initiated without heating, such as the reaction between sodium and carbon tetrachloride. Strategies based on these reactions have already been adopted to create pores in carbonaceous products. However, an appraisal of them is still lacking. In this mini review, we categorize these strategies into three types, namely, hydrothermal carbonization (HTC), *in situ* hard templating (HT) method, and mechanically induced self-sustaining reaction (MSR). These three types of strategies are discussed individually in the following sections. Finally, advantages and problems related to them are summarized. It is noteworthy that this review does not intend to cover conventional methods including post-synthesis activation and the soft templating method, which often require thermal treatment at temperatures above 600°C and have already been well discussed in the literature (Liang et al., 2008; Sevilla and Mokaya, 2014; Liu et al., 2015).

## 2 Hydrothermal carbonization

Hydrothermal carbonization is a type of the hydrothermal processing of materials. Like other hydrothermal processes, HTC is typically conducted in a steel pressure vessel known as an autoclave. At elevated temperatures (often in the range of 150°C–350°C), water in the autoclave is in a subcritical state. It acts as both a solvent and a catalyst for the fragmentation of a variety of starting substances, such as carbohydrates, lignin, and biomass (Jain et al., 2016). Next, the resultant small molecules undergo various reactions such as substitution, cycloaddition, and polymerization to form carbonaceous products. Meanwhile, large chunks of an insoluble starting material may still be present, and they become chars via solid-solid conversion involving extensive dehydration and condensation reactions. Carbonaceous materials from HTC (often termed as hydrochars) typically have low degrees of condensation and are decorated with large amounts of oxygenous functional groups. Additionally, they suffer from a major drawback of a lack of nanopores, resulting in small SSA values (typically below 50 m^2^/g) (Titirici et al., 2007b; Sevilla and Fuertes, 2009b; a; Titirici and Antonietti, 2010; Titirici et al., 2012; Yu et al., 2012; Falco et al., 2013b; Zhu et al., 2014). Therefore, HTC is often followed by post-synthesis activation at temperatures above 600°C to create primarily micropores (Yu et al., 2012; Falco et al., 2013a; Zhu et al., 2014; Chen et al., 2020; Yun et al., 2022).

**FIGURE 1:**
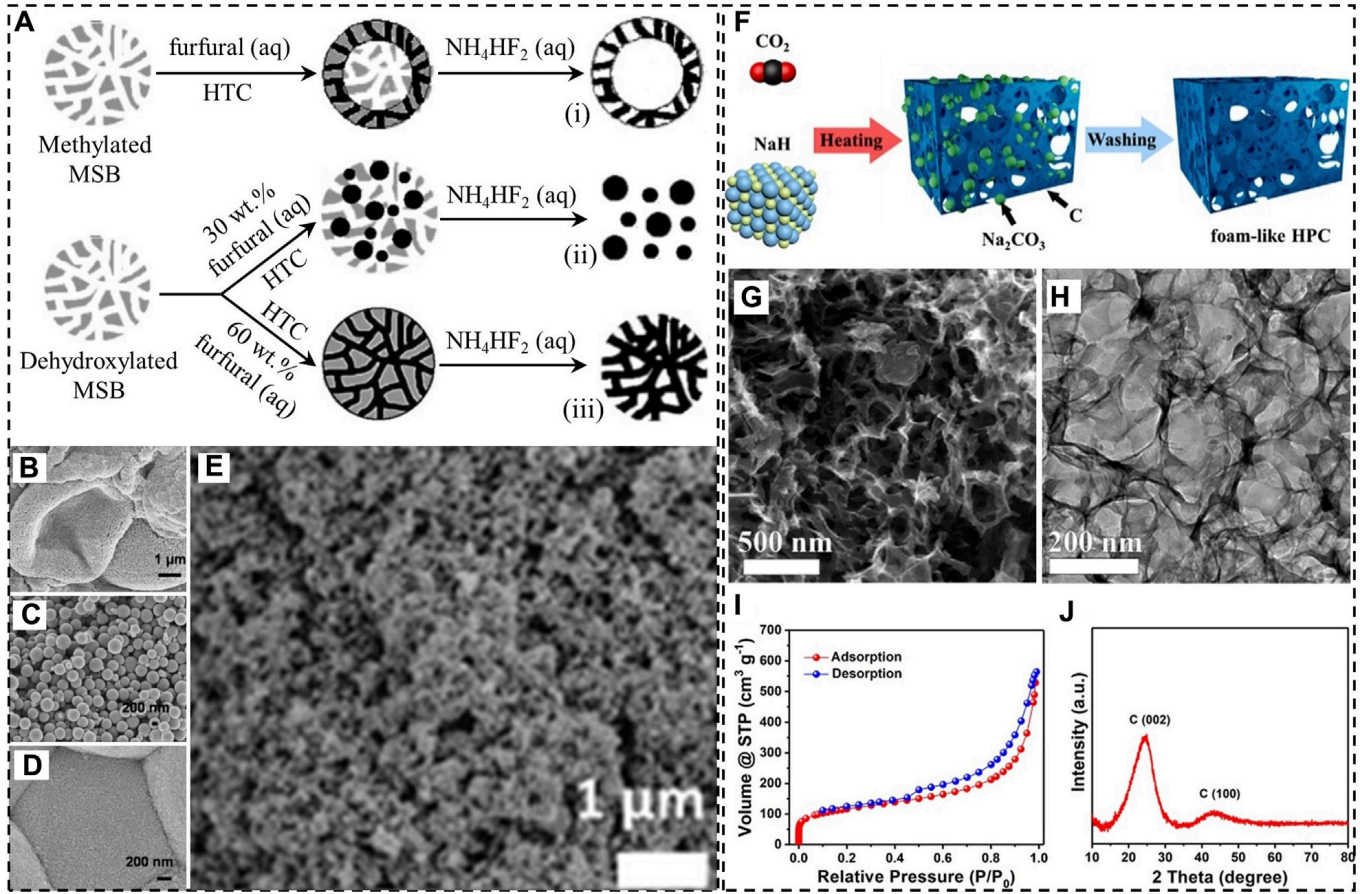
**(A)** Hydrothermal carbonization (HTC) of furfural in the presence of mesoporous silica beads (MSBs), **(B–D)** SEM images of the carbonaceous products (i-iii) in **A, (E)** SEM image of an aerogel-like carbonaceous material synthesized under hypersaline conditions; **(F)** synthesis of hierarchically porous carbon (HPC) by reacting CO_2_ with NaH, **(G)** SEM image, **(H)** TEM image, **(I)** N_2_ adsorption-desorption isotherm, **(J)** XRD pattern of HPC. **(A–D)** Adapted with permission from (Titirici et al., 2007a), Copyright 2007 Wiley-VCH; **(E)** reprinted with permission from (Fechler et al., 2013), Copyright 2013 Royal Society of Chemistry; **(F–J)** reprinted with permission from (Wang et al., 2018), Copyright 2018 American Chemical Society.

Titirici et al. (2007a) showed an alternative strategy to prepare mesoporous carbonaceous materials by combing HTC with the hard templating method. In their work, hydrothermal carbonization of furfural was carried out at 180°C for 24 h in the presence of mesoporous silica beads (Figure 1A). Thermal calcination was not necessary before silica removal. However, the structure of carbonaceous products was heavily influenced by the surface chemistry of the silica templates. The use of mesoporous silica microspheres modified with methyl groups (moderately hydrophobic) led to hollow mesoporous carbonaceous spheres as the aqueous solution of furfural mostly stayed close to the surface (Figure 1B). For moderately hydrophilic silica microspheres with siloxane groups, the structure also depends on the amount of furfural added. A 30 wt.% furfural solution resulted in carbonaceous microparticles with sizes considerably smaller than those of silica beads (Figure 1C), indicating that furfural failed to occupy the mesopores of the hard template. In contrast, mesoporous carbonaceous microspheres with a SSA of 200 m^2^/g were prepared when a 60 wt.% furfural solution was used (Figure 1D). Apparently, 200 m^2^/g is still far smaller than the SSA of activated carbon materials likely due to severe particle aggregation in hydrochars. Fechler et al. (2013) demonstrated that this uncontrollable aggregation of carbonaceous particles can be avoided by performing HTC in the presence of hygroscopic salt eutectics. The salts are able to stabilize the surface of carbonaceous nanoparticles and prevent them from growing excessively. As a result, the nanoparticles in different domains assemble into porous aerogel-like structures (Figure 1E). One problem of the strategy is the use of a great amount of salts, which are eventually removed using a large volume of water. For instance, to prepare such aerogel-like N-doped carbonaceous material with a SSA of 673 m^2^/g, 6 g of glucose as the carbon source and 1 g of 2-pyrrol-carboxyaldehyde as the nitrogen source were mixed with 15 g of the ZnCl_2_-LiCl eutectic and 3 g of water, followed by salt dissolution in 1 L of water.

## 3 *In situ* hard templating method

In a conventional hard templating process, a precursor is firstly introduced to the voids in a porous material (i.e., hard template), followed by thermal treatment that converts the precursor to a product and then template removal to generate porosity. In the modified hard templating method, a product and a template are produced simultaneously. Next, a porous structure can easily be created after dissolution of the *in situ* formed template without undergoing calcination.

Wang et al. (2018) proposed an *in situ* HT method to produce hierarchically porous carbon (HPC) by reacting CO_2_ with NaH at temperatures as low as 320°C in a glove box filled with Ar (Figure 1F). The X-ray diffraction (XRD) pattern of the reaction product discloses the existence of sodium carbonate. Hydrogen as a reaction product was detected using mass spectrometry (MS). Flow of H_2_ in the resultant carbon during the reaction as well as dissolution of Na_2_CO_3_ particles, which essentially functioned as a hard template, are both responsible for the formation of pores.

Scanning and transmission electron microscopy (SEM and TEM) images of HPC reveal a foam-like structure (Figures 1G, H). The presence of various types of pores, including micropores, mesopores, and macropores is evident from the N_2_ adsorption-desorption isotherm (Figure 1I), which shows large amounts of gas adsorbed at low relative pressures (p/p_0_), a hysteresis loop, and an unsaturated adsorption branch at p/p_0_ close to 1. SSA and the specific pore volume (SPV) were calculated to be 415 m^2^/g and 0.87 cm^3^/g, respectively. By applying density functional theory to the N_2_ adsorption-desorption data, two types of micropores with average sizes of 0.7 and 1.4 nm were identified. Nevertheless, the pore size distribution in the range above 4 nm is continuous and broad. Also, HPC is not graphitized according to the XRD pattern that shows two broad peaks at 24° and 44° (Figure 1J). Furthermore, a major concern of the synthesis is the usage of NaH, which reacts rapidly with water, generating heat and flammable hydrogen.

**FIGURE 2:**
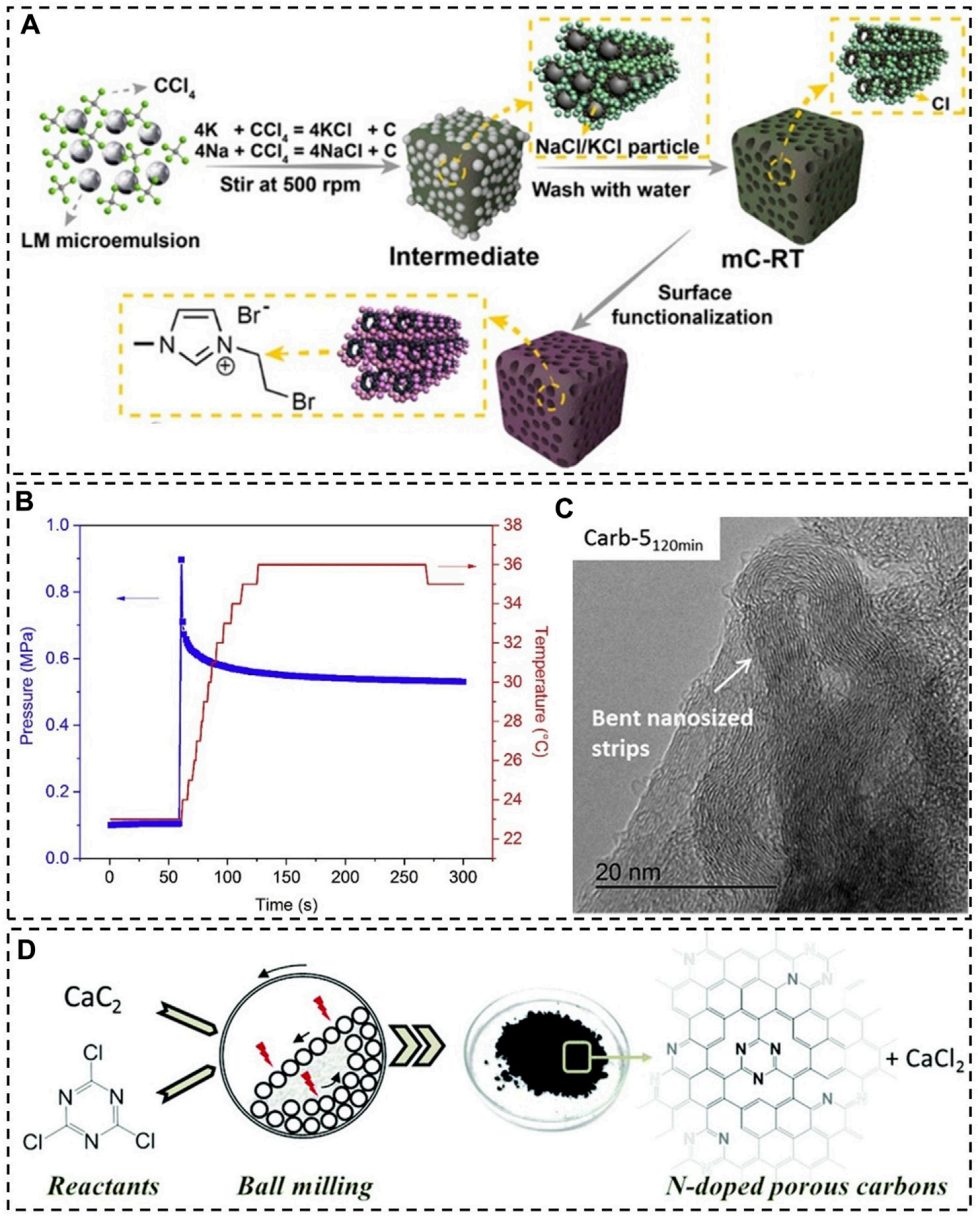
**(A)** Synthesis of a nanoporous carbon material (mC-RT) at room temperature, LM—liquid metal; **(B)** pressure and temperature evolution during the mechanically induced self-sustaining reaction between CaC_2_ and C_6_Cl_6_ in a ball mill, **(C)** TEM image of a reaction product; **(D)** mechanochemical synthesis of N-doped porous carbon materials. **(A)** Reprinted with permission from (Tang et al., 2022), Copyright 2022 Wiley-VCH; **(B,C)** reprinted with permission from (Casco et al., 2018), Copyright 2018 Elsevier; **(D)** reprinted with permission from (Casco et al., 2019), Copyright 2019 Royal Society of Chemistry.

Tang et al. (2022) reported a similar method that was carried out at room temperature for producing nanoporous carbon (Figure 2A). The synthesis occurred in the sodium-potassium alloy (NaK), which has a melting point of −12.7°C. Carbon tetrachloride (CCl_4_) was used as the carbon precursor and it was directly reduced by the NaK alloy into carbon, creating NaCl and KCl particles that were subsequently dissolved in water to introduce porosity. Porous carbon materials were produced from reactions with differing durations of 6, 12, 18, and 24 h. Their SSA was found to increase with extending reaction time. The largest SSA was assessed at 358 m^2^/g, which is relatively small compared to the SSA of activated carbon. N_2_ adsorption experiments also reveal the existence of both micropores (1.3–1.6 nm) and mesopores (2.8–8.7 nm) in the 24-h product (labeled as mC-RT) with a total volume of 0.36 cm^3^/g. However, due to the difficulty in controlling the size, shape and arrangement of NaCl and KCl particles formed, pores created in the carbon products have low degrees of uniformity and order. This problem has previously been noticed in the synthesis of porous carbon nitride materials using *in situ* formed templates (Kailasam et al., 2011; Zhang and Jaroniec, 2020).

Importantly, C-Cl bonds in the synthesized porous carbon materials allow for easy surface functionalization (Figure 2A). By heating mC-RT in a toluene-imidazole-trimethylamine mixture at 80°C for 24 h, its surface was functionalized with imidazole groups (the product is named as mC-RT-I). Further treatment of mC-RT-I in a toluene-dibromoethane mixture caused bromoethane to be attached to the imidazole groups, resulting in a product labeled as mC-RT-I/B. mC-RT-I/B turned out to be an effective catalyst for cycloaddition of CO_2_ to styrene oxide. Since mC-RT is unable to catalyze the reaction, it essentially acted as a catalyst support.

Like NaH, NaK also reacts with water to form hydrogen and hydroxides. For 1 g of NaK used, the theoretical yield of carbon is approximately 0.09 g. Taking into account the percentage yield of 52.2% reported, the actual yield of mC-RT was less than 0.05 g. In the meanwhile, 25 mL of toluene was necessary to disperse 1 g of NaK, which is likely to ramp up the cost and create additional waste. Moreover, CCl_4_ as the carbon source is highly toxic and environment-unfriendly, posing additional problem to the method.

Recently, Zhang et al. (2022) have demonstrated a reaction system that gave rise to amorphous porous carbonaceous nanoparticles (APCNs) at a low temperature of 180°C. The system consists of ferrocene, ammonium chloride, oxygen, and various organic solvents (e.g., phenoxyethanol, tetraglycol, glycol, etc.). The reaction is not susceptible to moisture and can safely be performed in a glass flask. A mixture of phenoxyethanol and glycol afforded APCNs with a large SSA of 936 m^2^/g. On the one hand, organic solvents were found to be crucial to achieve high SSA values as they improve the mixing of the reactants. On the other hand, their usage and recycling require extra money and time, especially considering the significant amounts of the organic solvents needed. For instance, in the preparation of APCNs with the largest SSA, 20 mL of glycol and 50 mL of phenoxyethanol were used, but only 0.14 g of the product was produced.

## 4 Mechanically induced self-sustaining reaction

Mechanochemical synthesis is typically conducted in ball mills without using solvents, generating minimum waste (Szczęśniak et al., 2020). Mechanochemistry has been applied for synthesizing porous carbonaceous materials for more than 2 decades (Chen et al., 1999; Salver-Disma et al., 1999). In the early stage, graphite was typically used as the starting material. Grinding of graphite in ball mills leads to a phase transformation from hexagonal to turbostratic at short times and amorphous at long times (Chen et al., 1999). In both turbostratic and amorphous carbons, the constituent graphene layers are distorted, giving rise to nanopores. In addition, ball milling of graphite creates carbon nanoparticles. Aggregation of these particles can also produce nanopores.

Recently, additional sources of carbon have been explored, including multi-walled carbon nanotubes (Soares et al., 2015), organic compounds (Zhang et al., 2018), biomass (Schneidermann et al., 2017; Schneidermann et al., 2019), among others. However, conversion of these precursors into carbon materials requires a carbonization step at elevated temperatures (often >600°C).

Casco et al. (2018) reported that this extra thermal process can be eliminated by taking advantage of the heat released from the grinding-induced reaction of starting materials (Casco et al., 2019), and it has been referred to as the mechanically induced self-sustaining reaction (MSR) (Mulas et al., 1997; Cao et al., 1999; Caschili et al., 2006). One of their works demonstrate a reaction between calcium carbide (CaC_2_) and hexachlorobenzene (C_6_Cl_6_) to produce nanoporous carbonaceous materials (Casco et al., 2018). Interestingly, the system remained at low temperatures (below 36°C) during the reaction in spite of the release of a large amount of heat (492 kcal) (Figure 2B). By ball milling a mixture of CaC_2_ and C_6_Cl_6_ at room temperature for a short time period of 5 min, a porous carbonaceous material with a SSA value of 475 m^2^/g was produced. Grinding for longer times seems to give rise to larger SSA values. The carbonaceous product obtained after grinding CaC_2_ and C_6_Cl_6_ with a mass ratio of 5.1 for 120 min has a SSA of 915 m^2^/g and a SPV of 0.96 cm^3^/g. TEM images show the presence of graphene layers in all the carbonaceous materials; however, after 120 min of grinding graphene layers became highly bent and distorted (Figure 2C). The formation of turbostratic carbon materials at short times (e.g., 5 min) is evidenced by X-ray scattering patterns. They gradually transformed into amorphous carbon with smaller electrical conductivity after further grinding (e.g., 120 min), resembling the case when graphite is used as the starting material.

Mechanically induced self-sustaining reactions (MSRs) have also been utilized to produce porous carbonaceous materials doped with heteroatoms at room temperature. Casco et al. (2019) reported the doping of porous carbon materials with N atoms by ball milling mixtures of calcium carbide and cyanuric chloride (Figure 2D). Elemental analysis of the resultant carbon materials shows that the N content in the products from a 120-min grinding process is consistently larger than that of the products from a 5-min grinding process, revealing two different doping mechanisms: *in situ* and *ex situ* doping. The former mechanism is associated with the reaction itself that generates N-containing carbon products. The latter mechanism arises from atom incorporation due to grinding. It is apparent that an increase in the mass ratio of CaC_2_ to C_3_Cl_3_N_3_ leads to a decline in the nitrogen content. Meanwhile, the existence of extra CaC_2_ benefits the formation of nanopores. Among the four CaC_2_-C_3_Cl_3_N_3_ mixtures studied with mass ratios of 0.5, 0.7, 2.3, and 4.6, the mixture with a ratio of 4.6 yielded a product with the largest SSA (1,080 m^2^/g) and SPV (1.25 cm^3^/g).

Unfortunately, MSRs for the synthesis of porous carbonaceous materials have been typically carried out in environments free of moisture due to the use of carbides, which can react with water, producing heat and flammable acetylene. Also, these reactions often result in a sudden increase in the pressure (Figure 2B). Some authors have pointed out that the reaction with large quantities of reactants at the stoichiometric ratio (e.g., 3 CaC_2_:1 C_6_Cl_6_) may lead to explosion. Indeed, precautionary measures must be taken with regard to all exothermic reactions performed in sealed ball mills.

## 5 Summary and outlook

As compared to the post-synthesis activation and conventional templating methods for producing nanoporous carbonaceous materials, hydrothermal carbonization (HTC), the *in situ* hard templating (HT) method, and mechanically induced self-sustaining reaction (MSR) have an apparent advantage, i.e., the requirement of less energy input. Particularly, MSR does not need solvents and can be carried out in a matter of minutes. Nonetheless, a few common problems with the current pyrolysis-absent synthetic strategies of nanoporous carbonaceous materials as discussed below need to be addressed.

(1) Relatively small specific surface areas. The specific surface area of hydrochars from HTC is typically below 200 m^2^/g. Nanoporous carbonaceous materials from *in situ* HT synthesis and MSR have SSA values up to 1,000 m^2^/g, which is still far smaller than that of sufficiently activated carbon [>3,000 m^2^/g (Dubey et al., 2020)]. A promising solution that integrates mechanochemical synthesis with chemical activation has been illustrated by Schneidermann et al. (2019). In their procedure, potassium carbonate as the activating agent was added into a mixture of non-purified wood waste (the carbon source), urea, and/or melamine (the N source). Eventually, nanoporous N-doped carbon materials with specific surface areas and pore volumes of up to 3,000 m^2^/g and 2 cm^3^/g, respectively, were obtained. These values are comparable to those of many activated carbon materials. However, the use of wood waste required additional calcination at 800°C in order to produce carbonaceous materials; precursors that can be carbonized at lower temperatures, such as hydroxymethylfurfural (Li et al., 2017), are recommended.
(2) Use of environment-unfriendly and/or pyrophoric substances. Many of the reactants as the carbon sources such as carbon tetrachloride, hexachlorobenzene, etc. are quite toxic to humans and wildlife. Additionally, the large amounts of organic solvents needed in the *in situ* HT method eventually become wastes. Their treatment and recycling in turn drive up the economic cost of the synthesis. Lastly, the compounds for reducing carbon sources into the element, such as alkali metals, metal hydrides, etc. easily react with the moisture in air to generate flammable gases. It is clear that further efforts are still needed to develop more sustainable and safer reaction systems. One of the systems shown to be promising is composed of carbon-containing compounds as the reducing agents and oxygen in the air as the oxidizing agent (Zhang et al., 2022).
(3) Broad pore size distribution curves. So far, either the *in situ* HT method or MSR alone is not able to generate uniform nanopores in carbonaceous materials. The pore sizes often cover the entire range of nanopores, namely, 1–100 nm. Zhang et al. (2018) demonstrated the preparation of carbonaceous materials with highly ordered nanoporous structures by combining mechanochemical synthesis with the hard templating method. They ground a mixture of copper chloride and bipyridine with the presence of silica nanoparticles in a ball mill. The grinding induced a coordination reaction between copper chloride and bipyridine, resulting in a polymer that subsequently filled the voids between silica nanoparticles. Next, a pyrolysis step at 500°C was necessary to convert the polymer into carbon. After removing the nanoparticles, an ordered mesoporous carbon material was produced as indicated by the small-angle XRD pattern.

As can be seen, all these issues are resolvable. Nonetheless, efforts are still necessary in order to achieve truly low-temperature synthesis of nanoporous carbonaceous materials with a good degree of pore uniformity.

## References

[B1] Cao G. Doppiu S. Monagheddu M. Orrù R. Sannia M. Cocco G. (1999). Thermal and mechanochemical self-propagating degradation of chloro-organic compounds: the case of hexachlorobenzene over calcium hydride. Ind. Eng. Chem. Res. 38, 3218–3224. 10.1021/ie980790+

[B2] Caschili S. Delogu F. Concas A. Pisu M. Cao G. (2006). Mechanically induced self-propagating reactions: analysis of reactive substrates and degradation of aromatic sulfonic pollutants. Chemosphere 63, 987–995. 10.1016/j.chemosphere.2005.08.052 16310824

[B3] Casco M. E. Badaczewski F. Grätz S. Tolosa A. Presser V. Smarsly B. M. (2018). Mechanochemical synthesis of porous carbon at room temperature with a highly ordered sp^2^ microstructure. Carbon 139, 325–333. 10.1016/j.carbon.2018.06.068

[B4] Casco M. E. Kirchhoff S. Leistenschneider D. Rauche M. Brunner E. Borchardt L. (2019). Mechanochemical synthesis of N-doped porous carbon at room temperature. Nanoscale 11, 4712–4718. 10.1039/c9nr01019j 30838363

[B5] Chen W. Zhang G. Li D. Ma S. Wang B. Jiang X. (2020). Preparation of nitrogen-doped porous carbon from waste polyurethane foam by hydrothermal carbonization for H_2_S adsorption. Ind. Eng. Chem. Res. 59, 7447–7456. 10.1021/acs.iecr.0c00498

[B6] Chen Y. Fitz Gerald J. Chadderton L. T. Chaffron L. (1999). Nanoporous carbon produced by ball milling. Appl. Phys. Lett. 74, 2782–2784. 10.1063/1.124012

[B7] Diez N. Sevilla M. Fuertes A. B. (2021). Synthesis strategies of templated porous carbons beyond the silica nanocasting technique. Carbon 178, 451–476. 10.1016/j.carbon.2021.03.029

[B8] Dubey P. Shrivastav V. Maheshwari P. H. Sundriyal S. (2020). Recent advances in biomass derived activated carbon electrodes for hybrid electrochemical capacitor applications: challenges and opportunities. Carbon 170, 1–29. 10.1016/j.carbon.2020.07.056

[B9] Falco C. Marco-Lozar J. P. Salinas-Torres D. Morallón E. Cazorla-Amorós D. Titirici M.-M. (2013a). Tailoring the porosity of chemically activated hydrothermal carbons: influence of the precursor and hydrothermal carbonization temperature. Carbon 62, 346–355. 10.1016/j.carbon.2013.06.017

[B10] Falco C. Sieben J. M. Brun N. Sevilla M. Van Der Mauelen T. Morallón E. (2013b). Hydrothermal carbons from hemicellulose‐derived aqueous hydrolysis products as electrode materials for supercapacitors. ChemSusChem 6, 374–382. 10.1002/cssc.201200817 23319452

[B11] Fechler N. Wohlgemuth S.-A. Jäker P. Antonietti M. (2013). Salt and sugar: direct synthesis of high surface area carbon materials at low temperatures via hydrothermal carbonization of glucose under hypersaline conditions. J. Mater. Chem. A 1, 9418–9421. 10.1039/c3ta10674h

[B12] Jain A. Balasubramanian R. Srinivasan M. (2016). Hydrothermal conversion of biomass waste to activated carbon with high porosity: a review. Chem. Eng. J. 283, 789–805. 10.1016/j.cej.2015.08.014

[B13] Kailasam K. Epping J. D. Thomas A. Losse S. Junge H. (2011). Mesoporous carbon nitride–silica composites by a combined sol–gel/thermal condensation approach and their application as photocatalysts. Energy Environ. Sci. 4, 4668–4674. 10.1039/c1ee02165f

[B14] Lee J. Kim J. Hyeon T. (2006). Recent progress in the synthesis of porous carbon materials. Adv. Mater. 18, 2073–2094. 10.1002/adma.200501576

[B15] Li S. Zhang H. Wang G. Liu L. Yu Y. Chen A. (2017). Biomass derived 5-hydroxymethylfurfural as carbon precursor to form hollow carbon nanospheres for CO_2_ capture. Fullerenes, nanotub. Carbon nanostruct. 25, 493–496. 10.1080/1536383x.2017.1342629

[B16] Liang C. Li Z. Dai S. (2008). Mesoporous carbon materials: synthesis and modification. Angew. Chem. Int. Ed. 47, 3696–3717. 10.1002/anie.200702046 18350530

[B17] Libbrecht W. Verberckmoes A. Thybaut J. W. Van Der Voort P. De Clercq J. (2017). Soft templated mesoporous carbons: tuning the porosity for the adsorption of large organic pollutants. Carbon 116, 528–546. 10.1016/j.carbon.2017.02.016

[B18] Liu J. Wickramaratne N. P. Qiao S. Z. Jaroniec M. (2015). Molecular-based design and emerging applications of nanoporous carbon spheres. Nat. Mater. 14, 763–774. 10.1038/nmat4317 26201892

[B19] Mulas G. Loiselle S. Schiffini L. Cocco G. (1997). The mechanochemical self-propagating reaction between hexachlorobenzene and calcium hydride. J. Solid State Chem. 129, 263–270. 10.1006/jssc.1996.7238

[B20] Salver-Disma F. Tarascon J.-M. Clinard C. Rouzaud J.-N. (1999). Transmission electron microscopy studies on carbon materials prepared by mechanical milling. Carbon 37, 1941–1959. 10.1016/s0008-6223(99)00059-7

[B21] Schneidermann C. Jäckel N. Oswald S. Giebeler L. Presser V. Borchardt L. (2017). Solvent‐free mechanochemical synthesis of nitrogen‐doped nanoporous carbon for electrochemical energy storage. ChemSusChem 10, 2416–2424. 10.1002/cssc.201700459 28436604

[B22] Schneidermann C. Kensy C. Otto P. Oswald S. Giebeler L. Leistenschneider D. (2019). Nitrogen‐doped biomass‐derived carbon formed by Mechanochemical synthesis for lithium–sulfur batteries. ChemSusChem 12, 310–319. 10.1002/cssc.201801997 30303617

[B23] Sevilla M. Fuertes A. B. (2009a). Chemical and structural properties of carbonaceous products obtained by hydrothermal carbonization of saccharides. Chem. Eur. J. 15, 4195–4203. 10.1002/chem.200802097 19248078

[B24] Sevilla M. Fuertes A. B. (2009b). The production of carbon materials by hydrothermal carbonization of cellulose. Carbon 47, 2281–2289. 10.1016/j.carbon.2009.04.026

[B25] Sevilla M. Mokaya R. (2014). Energy storage applications of activated carbons: supercapacitors and hydrogen storage. Energy Environ. Sci. 7, 1250–1280. 10.1039/c3ee43525c

[B26] Soares O. Rocha R. Gonçalves A. Figueiredo J. Órfão J. Pereira M. (2015). Easy method to prepare N-doped carbon nanotubes by ball milling. Carbon 91, 114–121. 10.1016/j.carbon.2015.04.050

[B27] Szczęśniak B. Borysiuk S. Choma J. Jaroniec M. (2020). Mechanochemical synthesis of highly porous materials. Mater. Horiz. 7, 1457–1473. 10.1039/d0mh00081g

[B28] Tang D. Wang T. Zhang W. Zhao Z. Zhang L. Qiao Z. A. (2022). Liquid Na/K alloy interfacial synthesis of functional porous carbon at ambient temperature. Angew. Chem. Int. Ed. 61, e202203967. 10.1002/anie.202203967 35471735

[B29] Titirici M.-M. Antonietti M. (2010). Chemistry and materials options of sustainable carbon materials made by hydrothermal carbonization. Chem. Soc. Rev. 39, 103–116. 10.1039/b819318p 20023841

[B30] Titirici M.-M. White R. J. Falco C. Sevilla M. (2012). Black perspectives for a green future: hydrothermal carbons for environment protection and energy storage. Energy Environ. Sci. 5, 6796–6822. 10.1039/c2ee21166a

[B31] Titirici M. M. Thomas A. Antonietti M. (2007a). Replication and coating of silica templates by hydrothermal carbonization. Adv. Funct. Mater. 17, 1010–1018. 10.1002/adfm.200600501

[B32] Titirici M. M. Thomas A. Yu S.-H. Müller J.-O. Antonietti M. (2007b). A direct synthesis of mesoporous carbons with bicontinuous pore morphology from crude plant material by hydrothermal carbonization. Chem. Mater. 19, 4205–4212. 10.1021/cm0707408

[B33] Wang H. Shao Y. Mei S. Lu Y. Zhang M. Sun J.-K. (2020). Polymer-derived heteroatom-doped porous carbon materials. Chem. Rev. 120, 9363–9419. 10.1021/acs.chemrev.0c00080 32786418

[B34] Wang K. Feng G. Liang C. Xia Y. Zhang J. Gan Y. (2018). Green and low-temperature synthesis of foam-like hierarchical porous carbon from CO_2_ as superior lithium storage material. ACS Appl. Energy Mater. 1, 7123–7129. 10.1021/acsaem.8b01549

[B35] Xie L. Jin Z. Dai Z. Chang Y. Jiang X. Wang H. (2020). Porous carbons synthesized by templating approach from fluid precursors and their applications in environment and energy storage: a review. Carbon 170, 100–118. 10.1016/j.carbon.2020.07.034

[B36] Yu L. Falco C. Weber J. White R. J. Howe J. Y. Titirici M.-M. (2012). Carbohydrate-derived hydrothermal carbons: a thorough characterization study. Langmuir 28, 12373–12383. 10.1021/la3024277 22853745

[B37] Yun H. Kim Y. J. Kim S. B. Yoon H. J. Kwak S. K. Lee K. B. (2022). Preparation of copper-loaded porous carbons through hydrothermal carbonization and ZnCl_2_ activation and their application to selective CO adsorption: experimental and DFT calculation studies. J. Hazard. Mater. 426, 127816. 10.1016/j.jhazmat.2021.127816 34865899

[B38] Zhang E. Hao G.-P. Casco M. E. Bon V. Grätz S. Borchardt L. (2018). Nanocasting in ball mills–combining ultra-hydrophilicity and ordered mesoporosity in carbon materials. J. Mater. Chem. A 6, 859–865. 10.1039/c7ta10783h

[B39] Zhang F. Liu B. Ni Z. Zhang X. Shao Y. Zhang F. (2022). Low-temperature organic solvent-based synthesis of amorphous porous carbon nanoparticles with high specific surface area at ambient atmosphere. Carbon 200, 281–295. 10.1016/j.carbon.2022.08.057

[B40] Zhang L. Jaroniec M. (2020). Strategies for development of nanoporous materials with 2D building units. Chem. Soc. Rev. 49, 6039–6055. 10.1039/d0cs00185f 32692344

[B41] Zhu X. Liu Y. Zhou C. Luo G. Zhang S. Chen J. (2014). A novel porous carbon derived from hydrothermal carbon for efficient adsorption of tetracycline. Carbon 77, 627–636. 10.1016/j.carbon.2014.05.067

